# Policy Goals of Contract Arrangements in Primary Care in Jeopardy: A Cross-Sectional Consumer Satisfaction Survey of Community Residents in Hangzhou, China

**DOI:** 10.3389/fpubh.2022.800612

**Published:** 2022-05-02

**Authors:** Lixian Ren, Jianping Ren, Chaojie Liu, Mengyan He, Xiantao Qiu

**Affiliations:** ^1^Department of Health Management, School of Public Health, Hangzhou Normal University, Hangzhou, China; ^2^Department of Public Health, School of Psychology and Public Health, La Trobe University, Melbourne, VIC, Australia

**Keywords:** primary care, consumer satisfaction, care contract, general practitioners, healthcare needs

## Abstract

**Objective:**

China is attempting to establish a voluntary contracting system in primary care. This study aimed to determine the degree of consumer satisfaction with the entitlements of contract arrangements and its associated factors.

**Methods:**

A stratified cluster sampling strategy was adopted to recruit study participants from three administrative districts of Hangzhou municipality, each containing six residential communities. In each community, 50 households were recruited. A questionnaire was administered through face-to-face interviews with members of the households who signed a contract with community health centers, to collect data regarding their sociodemographic characteristics, health conditions, and knowledge of and attitudes toward the contract entitlements. Consumer satisfaction was measured using six items rated on a five-point Likert scale ranging from “1=very dissatisfied” to “5=very satisfied” and a summed score was calculated. A mixed linear regression model was established to identify individual predictors of consumer satisfaction after adjustment of the random (intercept) effect of household clusters.

**Results:**

Overall, the respondents reported low levels of awareness and understanding of the contract entitlements, with an average knowledge score of 8.21 (SD = 3.74) out of a maximum possible of 19. The respondents had relatively lower levels of satisfaction (satisfied or very satisfied) with their prioritized entitlements (51.5%) and hospitals at home and telemedicine services (31.3%), compared with the contract and insurance policies (85.5%) and medical services provided (87.0%). Female gender, older age, chronic conditions, and perceived better health were associated with higher levels of satisfaction, while poor awareness and knowledge were associated with lower levels of satisfaction.

**Conclusion:**

The study participants perceived limited benefits from the contract arrangements in primary care, which may jeopardize the policy purpose of the arrangements to encourage patients to use primary care as the first contact point in accessing health care services. It is evident that consumer satisfaction with the contract entitlements varies by healthcare needs. Lower levels of satisfaction are associated with poor awareness and knowledge of the entitlements.

## Background

Internationally, there is consensus that 90% of health problems can be appropriately managed in primary care settings, while the rest require specialist consultations (1). Since the 1960s, the important role and functions of general practitioners (also known as family doctors in some countries) in the development of an efficient and quality health system have been increasingly recognized. They are the backbone of the primary care sector, aiming at maintaining and promoting both individual and population health. Empirical evidence shows that a primary-care-dominant health system outperforms the others in population health outcomes (2).

In a primary-care-dominated health care system, general practice is usually a predominant stream of medical specialization. General practitioners (GPs) are often assigned a gate-keeping role and serve as the first contact point for consumers to access health care services. Some countries even go further by introducing contract arrangements to ensure that GPs also look after the entire group of patients, not just individuals. The UK national health system (NHS) is deemed an exemplar (3). It is a publicly-funded system in which each GP is responsible for managing about 2,000 contracted patients. Specialist consultations and hospital services except for emergency care require a referral from the GPs (4). Contract arrangements have the advantage of promoting continuity and coordination of patient care despite some compromise to patient freedom of choice (2). Evidence shows that the UK system is characterized by a relatively low investment with high returns (5). Under such a system, GPs attach great importance to preventive services (6). Despite the stark contrast with the UK system, in the largely market-driven health system in the US, contract arrangements have also demonstrated their potential to reduce hospitalization and readmission of hospital patients (7), although it is often considered as a cost containment measure (8, 9).

Many countries have not introduced a contract system for various reasons. Germany, for example, has a strong tradition of corporatism and consumers enjoy freedom in choosing their first contact doctors, as well as in negotiating referred doctors (8). Contract arrangements are usually absent in low- and middle-income countries simply because they do not have enough skilled primary care professionals. This has resulted in a serious shortage of research into the potential effects of contract arrangements in low- and middle-income countries (10).

China is an upper middle-income country. Over the past two decades, China has been attempting to establish a GP-led health system. However, general practice is still in its early stage of development. Despite a serious shortage of GPs, there is both an under-use of primary care facilities and an over-use of hospital facilities in China (11, 12). The freedom of patients to bypass primary care in seeking hospital services is often blamed for the irrational distribution of health resources and services. For historical reasons, primary care facilities in China employ a large number of physicians without a university medical degree. Concerns over quality have deterred the Chinese government from introducing a gate-keeping and referral mechanism similar to the UK and several other countries (13). As an alternative option, a voluntary contracting system was introduced (14). In May 2016, the national government issued guidelines to promote primary care contract arrangements. Local governments were also encouraged to develop and use contract entitlements to incentivize patients to increase their use of primary care facilities (15). These may involve certain free services, prioritized medical appointments and referrals, and favorable policies for social health insurance programs (16). Although the contract is usually signed between a GP and a patient, the entitlements go beyond the scope of the services delivered by the GP (14).

The existing studies on China have focused on documenting the coverage of the contract arrangements (14), with a prior assumption that such arrangements are beneficial to the patients and the health care system. However, little is known about the experience of the community residents who enter into the contracts. This study contributes to the literature by measuring the satisfaction of those who signed a contract with their local community health centers. Internationally, measuring consumer satisfaction has become an important step for quality improvement (17).

## Methods

A cross-sectional questionnaire survey of community residents was conducted in Hangzhou. Ethics approval was obtained from Hangzhou Normal University (Reference number 20190070). The survey was anonymous and oral informed consent was sought from the study participants prior to the survey.

### Study Setting

Hangzhou is the capital city of Zhejiang province, one of the most developed regions in China. More than 10 million people reside in Hangzhou permanently and about 23% are older than 60 years. There are 5,925 health institutions in Hangzhou, including 2,626 community health centers (CHCs) (18). Community health services are available to all Hangzhou residents, including voluntary contract arrangements with local CHCs. Hangzhou started the voluntary contract initiative in 2014 and scaled it up across all CHCs in 2016. A total of 40.551 million patient visits to CHCs were recorded in Hangzhou in 2019 (18).

### Sampling and Data Collection

A multi-stage stratified sampling strategy was adopted. Administrative districts were sorted in order according to their local gross domestic products and evenly divided into high-, middle-, and low-income districts. One district in each category was selected: Xiacheng (high income), Jianggan (middle income), and Gongshu (low income). Six residential communities were then conveniently selected from each participating district. In each residential community, 50 households were randomly selected. All adult (>18 years) household members were eligible to participate in the study. The households that rejected invitations or were not approachable were replaced by neighboring households.

Data were collected through household visits and face-to-face interviews in November 2018. Interviewers were recruited from local CHC health workers. A total of 36 interviewers were trained and grouped into six teams. Each team visited one residential community at a time. Each eligible household member was interviewed separately and completed the questionnaire independently. Those who were absent or refused to give informed consent were excluded. This resulted in a final sample of 2,315 individuals from 906 households. After excluding those who did not enter into a contract arrangement with local CHCs, 1,304 (56.33%) respondents from 673 households were included in data analyses for the purpose of this study. This sample size was at least 25 times the number of independent variables for regression modeling, far exceeding the minimal requirement (19).

### Measurements

Satisfaction was defined as a subjective perception, reflecting the gap between one's expectation and actual experience (20). It was measured using a six-item scale in this study, covering six aspects of the contract entitlements, namely (1) favorable contract and insurance policies; (2) free services covered by the contract; (3) free personal inquiries and appointments; (4) prioritized consultations and referral; (5) hospitals at home and telemedicine; and (6) free prescription services for chronic diseases. The participants were asked to rate their degree of satisfaction with each of the six items on a five-point Likert scale, ranging from 1 (very dissatisfied) to 5 (very satisfied). Satisfaction ratings on the six items were summed for each respondent. The respondents were then divided into two groups using a cut-off point of 24 (exceeding the neutral total): low satisfaction (<24) and high satisfaction (≥24).

Knowledge has a significant impact on individual expectations (21). In this study, an eleven-item scale was developed to measure awareness of the respondents about their contracting institution (item 1) and GPs (item 2), and the benefits they were entitled to in line with the contract policy, which included discounts in out-of-pocket payments for insurance-covered services delivered in the contracting institution (300 Yuan reduction in deductibles–item three; 3% increase in reimbursement rates–item four); prioritized (free of charge) medical appointments (item five), consultations (item six) and referrals (item seven); free access to inquiries on personal records (item eight); and free prescribing services for chronic diseases (item nine). A score of one was assigned to a correct answer or a recognized benefit; 0 was recorded otherwise (including answer of unsure). The respondents were then asked to rate their overall understanding of the contract arrangements (item 10) and their entitlement to free prescribing services for chronic diseases (item 11) on a five-point Likert scale, ranging from 1 (very poorly) to 5 (very well). A summed score (ranging from 2 to 19) for each respondent was calculated. The respondents were subsequently divided into three knowledge groups: low (2–6, the lowest one quarter of the score range), medium (7–10, the lower middle range of score) and high (11–19, the higher middle and upper range of score), for the purpose of regression analyses since no linear correlation between knowledge and satisfaction could be assumed.

Health insurance benefits are an important component of the contract entitlement package. Although China has achieved almost universal coverage of social health insurance, multiple insurance programs with great disparities exist (22). In this study, we asked the respondents to report their membership of the two major social insurance programs: basic medical insurance for urban employees (BMIUE) and basic medical insurance for urban/rural residents (BMIUR). Both insurance programs require individual monetary contributions through premiums and copayments. The BMIUE usually has a larger funding pool and offers more generous financial benefits to enrollees than the BMIUR (23).

Previous research shows that consumer satisfaction with health care services often varies by sociodemographic characteristics of the consumers (20). In this study, we collected data in relation to the respondents' age (years), gender (male, female), marital status (unmarried, married, widowed), education (≤ primary school, middle school, high school, ≥university), employment status (employed, retired, unemployed), and occupation (management, professional, office clerical staff, company sales and services, self-employed, industrial worker, farming, other). We also asked the respondents to report their health status measured by chronic diseases (yes, no) and self-rated general health (poor, good, excellent).

### Data Analyses

The characteristics of the study participants were described through frequency distributions. Their knowledge of the contract arrangements (with a normal distribution) was described using mean scores and standard deviations, as well as percentage distributions across the high, medium and low knowledge groups.

Consumer satisfaction was described using mean and standard deviation of the satisfaction score (with a normal distribution), and the percentage of high satisfaction. Student *t*-tests (or ANOVA) and chi-squared tests were performed to identify the differences in the satisfaction score and the level of satisfaction, respectively, among respondents with different characteristics. The household cluster effect on consumer satisfaction was significant (ICC > 0.8). Therefore, a mixed linear regression model was established to determine individual predictors of patient satisfaction after adjustment of the random effect (intercept) of the family clusters. Independent variables were entered into the model in a block (enter approach) and all were coded as categorical measurements.

Data were managed using Epidata 3.1. Double data entry was conducted to ensure the accuracy of the data. Statistical analyses were performed using SPSS 21.0 software. A *p* < 0.05 was considered statistically significant.

## Results

### Characteristics of Respondents

Slightly over half (54%) of the study participants were women. The respondents had a mean age of 59 years: more than 60% were older than 60 years. The majority had not completed high school (62%), were married (86%) and retired (71%) at the time of the survey. More than 53% of respondents reported chronic diseases, but most self-rated their health as good (49%) or excellent (28%) (Table 1).

**Table 1:** Characteristics of study participants.

**Characteristics**	**Number**	**Percentage**
	**(Person)**	**(%)**
**Sex**		
Male	596	46.0
Female	701	54.0
**Age (years)**		
<18	90	6.9
18–40	126	9.7
41–60	290	24.0
>60	791	61.0
**Educational attainment**		
≤ Primary school	375	31.0
Middle school	372	30.8
High school	271	22.4
≥ University	191	15.8
**Employment**		
Employed	272	22.4
Retired	862	71.0
Unemployed	80	6.6
**Occupation**		
Management	188	16.9
Professional	195	17.5
Office clerical staff	202	18.1
Company sales and services	123	11.0
Self-employed	55	4.9
Industrial workers	171	15.3
Farming	80	7.2
Others	101	9.1
**Marital status**		
Unmarried	63	5.3
Married	1,028	86.1
Widowed	103	8.6
**Basic medical insurance for employees**		
Yes	614	47.4
No	682	52.6
**Basic medical insurance for residents**		
Yes	661	51.0
No	635	49.0
**Chronic disease**		
Yes	699	56.4
No	541	43.6
**Self-rating on health**		
Poor	216	23.3
Good	456	49.2
Excellent	255	27.5

### Knowledge About Contract Entitlements

The respondents had low levels of knowledge about their contract entitlements (Table 2). More than 72.8% fell into the low (34.0%) and medium (38.7%) knowledge groups, resulting in an average score of 8.21 (SD = 3.74) out of a maximum possible of 19. Less than half of the respondents could name their contracting doctors (38.2%) or institutions (34.5%) correctly. About 59.4% only identified one or two benefits to which they were entitled. The most noticeable benefit was the 300 Yuan (US$50) reduction in deductibles for insurance-covered outpatient services (54.4%), followed by prioritized medical appointments (29.6%), 3% increase in insurance reimbursement rates (26.1%), and free access to inquiries on personal records (26.1%). Most respondents (56%) were not aware of the free prescribing services for chronic diseases.

**Table 2:** Knowledge of study participants about contract arrangements.

**Study participants**	**Knowledge about contract arrangements**					
	**($\bar{x}\pm$SD)**	***p***	***% Low***	***% Medium***	***% High***	***p***
**Sex**		0.216				0.509
Male	8.05 ± 3.78		46.9	43.6	42.8	
Female	8.34 ± 3.72		53.1	56.4	57.2	
**Age (years)**		<0.001				<0.001
<18	3.41 ± 2.76		10.1	1.1	0.3	
18–40	7.37 ± 4.23		12.7	4.8	7.9	
41–60	8.16 ± 3.70		23.5	21.1	22.1	
>60	8.64 ± 3.50		53.7	72.9	69.6	
**Educational attainment**		0.015				<0.001
≤ Primary school	7.79 ± 3.23		36.7	34.0	21.1	
Middle school	8.93 ± 3.56		22.3	33.8	35.3	
High school	8.61 ± 3.81		23.3	19.1	27.7	
≥ University	8.34 ± 4.18		17.5	13.1	15.8	
**Employment**		<0.001				<0.001
Employed	7.74 ± 4.19		27.9	14.9	20.0	
Retired	8.68 ± 3.47		63.4	80.5	76.4	
Unemployed	7.26 ± 3.33		8.7	4.7	3.6	
**Occupation**		0.030				<0.001
Management	8.96 ± 3.84		16.2	16.5	21.7	
Professional	8.61 ± 3.88		16.9	17.0	19.3	
Office clerical staff	8.52 ± 3.94		18.9	15.6	22.4	
Company sales and services	8.01 ± 3.73		12.6	7.9	11.2	
Self-employed	7.40 ± 3.08		4.0	4.7	1.4	
Industrial workers	8.51 ± 3.44		12.9	19.5	15.6	
Farming	7.30 ± 2.89		8.9	9.4	2.7	
Others	8.15 ± 3.70		9.6	9.4	5.8	
**Marital status**		0.026				0.003
Unmarried	6.98 ± 4.14		8.3	2.6	3.9	
Married	8.38 ± 3.72		82.4	87.6	89.2	
Widowed	8.03 ± 3.48		9.2	9.7	6.9	
**Basic medical insurance for employees**		<0.001				<0.001
Yes	9.07 ± 3.60		38.1	48.3	65.0	
No	7.45 ± 3.67		61.9	51.7	35.0	
**Basic medical insurance for residents**		<0.001				<0.001
Yes	7.39 ± 3.63		60.3	51.3	31.7	
No	9.07 ± 3.63		39.7	48.7	68.3	
**Chronic disease**		<0.001				<0.001
Yes	9.17 ± 3.37		42.9	65.7	71.1	
No	7.30 ± 3.71		57.1	34.3	28.9	
**Self-rating on health**		0.023				0.010
Poor	9.29 ± 2.84		20.2	25.9	21.7	
Good	9.50 ± 3.12		43.4	48.0	54.7	
Excellent	8.85 ± 3.02		36.4	26.1	23.7	

### Consumer Satisfaction With Contract Arrangements

Overall, the respondents had a mean satisfaction score of 22.24 (SD = 3.98), falling short of the cut-off point of 24. Variations in satisfaction with the different aspects of contract arrangements were evident. The respondents reported relatively lower levels of satisfaction with prioritized entitlements (51.5%, *p* < 0.001) and hospitals at home and telemedicine services (31.3%, *p* < 0.001), compared with favorable contract and insurance policies (85.5%) and fee discounts in the medical services provided (87.0%). The mean scores on prioritized entitlements and hospitals at home and telemedicine services fell below 4 (satisfied) (Table 3).

**Table 3:** Consumer satisfaction with contract arrangements.

**Contract entitlement**	**Satisfaction rating**		
	**Mean ±SD**	**% of satisfied (≥4)**	**Ranking**
(1) Favorable contract and insurance policies	4.25 ± 0.77	85.5	2
(2) Fee discounts for medical services provided	4.32 ± 0.76	87.0	1
(3) Free personal inquiry and appointments	4.00 ± 0.82	70.9	4
(4) Prioritized consultations and referrals	3.71 ± 0.84	51.5	5
(5) Hospitals at home and telemedicine	3.41 ± 0.72	31.3	6
(6) Free prescriptions for chronic diseases	4.09 ± 0.81	79.0	3

### Factors Associated With Consumer Satisfaction With Contract Arrangements

The respondents who were older, retired, reported chronic conditions, and had a higher knowledge score on contract entitlements reported significantly (*p* < 0.001) higher degrees of satisfaction than the others. Similarly, those respondents were also more likely to fall into the high satisfaction group. Women were more likely to report higher satisfaction than men (Table 4).

**Table 4:** Satisfaction with contract arrangements of respondents with different characteristics.

**Characteristics**	**Satisfaction score**			**High satisfaction (≥24)**	
	**$\bar{x}\pm$SD**	***F***	***p***	**%**	***p***
**Gender**		1.451	0.229		0.016
Male	19.79 ± 5.812			36.0	
Female	20.25 ± 6.228			64.0	
**Age (years)**		10.283	<0.001		0.006
<18	19.50 ± 3.082			0.4	
18–40	20.12 ± 3.384			4.1	
41–60	21.24 ± 4.033			17.8	
>60	22.84 ± 3.875			77.6	
**Educational attainment**		1.128	0.337		0.781
≤ Primary school	22.75 ± 3.724			33.6	
Middle school	22.27 ± 4.148			30.7	
High school	21.98 ± 4.235			21.2	
≥University	21.78 ± 3.640			14.5	
**Employment**		8.294	<0.001		0.021
Employed	20.65 ± 3.789			12.8	
Retired	22.72 ± 3.912			83.5	
Unemployed	21.55 ± 4.123			3.7	
**Occupation**		1.052	0.393		0.396
Management	19.00 ± 6.218			19.7	
Professional	19.25 ± 5.806			17.5	
Office clerical staff	18.19 ± 6.251			15.4	
Company sales and services	17.51 ± 6.861			8.1	
Self-employed	18.93 ± 6.353			4.3	
Industrial workers	19.22 ± 5.520			15.4	
Farming	18.31 ± 5.563			9.0	
Others	18.93 ± 6.439			10.7	
**Marital status**		2.279	0.103		0.071
Unmarried	20.67 ± 3.247			1.3	
Married	22.31 ± 4.019			89.2	
Widowed	22.87 ± 3.672			9.6	
**Basic medical insurance for urban employees**		1.225	0.269		0.381
Yes	22.25 ± 4.097			49.8	
No	22.23 ± 3.857			50.2	
**Basic medical insurance for residents**		0.613	0.434		0.836
Yes	22.18 ± 3.748			46.5	
No	22.29 ± 4.174			53.5	
**Chronic disease**		131.035	<0.001		<0.001
Yes	23.84 ± 3.425			84.0	
No	19.44 ± 3.288			16.0	
**Self-rating on health**		0.128	0.880		0.698
Poor	22.27 ± 3.814			23.9	
Good	22.44 ± 4.058			50.8	
Excellent	22.00 ± 3.843			25.2	
**Knowledge score about contract arrangements**		56.856	<0.001		<0.001
Low (1–6)	19.48 ± 3.578			8.2	
Medium (7–10)	22.13 ± 3.863			44.9	
High (≥11)	23.72 ± 3.604			46.9	
**Total**	22.24 ± 3.98			26.7	

The high ICC values of the null model indicated a significant cluster effect of households on consumer satisfaction. The two-level mixed effect linear model generated consistent results after adjustment for variations in the random effect of family clusters (*p* < 0.001) as compared with those of the individual-level fixed effect model. High levels of knowledge on contract entitlements (β = 2.184–4.858, *p* < 0.01) and self-rated excellent health (β = 0.980, *p* = 0.016) were associated with higher levels of satisfaction with contract arrangements, while the absence of chronic conditions (β = −4.034, *p* < 0.001) was associated with lower levels of satisfaction with contract arrangements (Table 5).

**Table 5:** Multivariate linear regression analysis on factors associated with consumer satisfaction.

**Predictor**	**Null model**	**Fixed effect model** (***R***^**2**^ **=** **0.231)**		**Mixed effect model**	
		**β**	**95% CI**	**β**	**95% CI**
**Gender (reference=Male)**					
Female		0.803	(0.015, 1.592)	0.354	(−0.016, 0.725)
**Age (reference** **=** **0–40 years)**					
41–60		−0.762	(−2.714, 1.190)	−0.436	(−1.718, 0.846)
>60		0.558	(−1.682, 2.798)	0.270	(−1.363, 1.903)
**Educational attainment (reference** ** = ≤ ** **Primary school )**					
Middle school		0.140	(−0.862, 1.141)	0.089	(−0.566, 0.743)
High school		−0.453	(−1.583, 0.676)	−0.492	(−1.278, 0.293)
≥University		0.666	(−0.798, 2.131)	0.076	(−0.923, 1.075)
**Employment (reference=Employed)**					
Retired		−0.572	(−2.062, 0.918)	−0.065	(−1.056, 0.926)
Unemployed		0.527	(−3.630, 4.684)	0.670	(−1.802, 3.142)
**Occupation (reference=Management)**					
Professionals		0.596	(−0.650, 1.842)	−0.497	(−1.409, 0.416)
Office clerical		0.017	(−1.236, 1.270)	−0.598	(−1.519, 0.324)
Commercial/services		−0.263	(−1.769, 1.243)	−0.502	(−1.523, 0.519)
Self employed		1.662	(−0.579, 3.904)	−0.368	(−2.094, 1.358)
Industrial workers		0.894	(−0.380, 2.169)	−0.044	(−1.015, 0.926)
Farmer		0.076	(−1.644, 1.795)	−0.192	(−1.655, 1.272)
Others		1.385	(−0.180, 2.949)	0.632	(−0.567, 1.832)
**Marital status (reference=Unmarried)**					
Married		0.765	(−1.305, 2.835)	−0.036	(−1.339, 1.267)
Widowed		0.102	(−2.341, 2.545)	0.034	(−1.606, 1.674)
**Basic medical insurance for urban employees (reference=Yes)**					
No		0.283	(−1.463, 2.029)	0.496	(−1.114, 2.106)
**Baisc medical insurance for residents (reference=Yes)**					
No		−1.183	(−2.945, 0.579)	−0.698	(−2.344, 0.947)
**Chronic conditions (reference=Yes)**					
No		−4.426	(−5.326, −3.525)	−4.034	(−4.598, −3.469)
**Self-assessment of health (reference=Poor)**					
Good		0.335	(−0.589, 1.259)	0.190	(−0.447, 0.827)
Excellent		1.561	(0.469, 2.653)	0.980	(0.183, 1.776)
**Knowledge about contract arrangements (reference=Low)**					
Medium		2.656	(1.623, 3.688)	2.184	(1.232, 3.137)
High		5.353	(4.228, 6.477)	4.858	(3.815, 5.901)
**intra-level correlation coefficient (ICC)**	0.80			0.88	
**Akaike Information Criterion (AIC)**	5,621			4,732	
**Schwarz's Bayesian Criterion (BIC)**	5,635			4,741	

## Discussion

The level of awareness and knowledge of contract entitlements of the study participants was very low, although they had all entered into a contract arrangement with their local CHC and the system has been fully implemented since 2016 (24). Less than half of the respondents could correctly identify two or more entitlements. The satisfaction rates with the contract entitlements ranged from 31 to 87%. Higher awareness, better perceived health, and living with chronic conditions are significant predictors of higher levels of satisfaction after adjustment for variations in other variables and the random effect of family clusters.

The low level of awareness and knowledge of the contract entitlements is concerning as it can jeopardize the attainment of the intended goals. We found that the financial benefit of discounts in out-of-pocket payments was most acknowledged, with 54.4% of respondents noticing the sizable 300 Yuan reduction in deductibles. Similar financial benefits are widely available across China despite variations in policy designs. In Shanghai, for example, contracted patients could be prescribed up to 2 months of medications for chronic conditions which saves costs on medical appointments and consultation fees, a benefit which is not readily available to non-contracted patients (25). However, it is not clear whether these financial benefits provide sufficient incentives to encourage consumers to seek medical attention from primary care facilities. Over the past decade, the volume of patient visits to primary care facilities has increased significantly; but patient visits to primary care as a proportion of the total volume of outpatient services has actually been declining in China (26). It appears that a lack of trust in primary care imposes a great barrier to diverting hospital patients to primary care. The perceived quality of medical services in primary care is often deemed inferior to those provided in hospitals (27). Although primary care has the advantage of maintaining continuous relationships with patients and GPs are well positioned to coordinate care on behalf of their patients (2), these benefits are not well endorsed by the patients, according to the findings of this study. Indeed, <30% of the participants in our study realized the benefits of prioritized medical appointments and referrals and free access to inquiries on personal records. Previous studies showed that many people do not trust the competency of primary care providers in disease diagnosis and management. The legacy of the barefoot doctors led them to believe that primary care doctors nowadays are still inferior to hospital specialists (28).

In this study, we found that 56.33% of Hangzhou residents entered into contract arrangements with local CHCs, which is slightly higher than the average of 50.43% in China (29). Although Hangzhou did not lead in the contract rate (30), it outperformed some other developed regions in China such as Shanghai Changning District (52.8%) (31) and Guangdong province (52.9%) (32). Perceived financial benefits may be a major contributor to encourage consumers to sign a contract with CHCs in China. We found that the contracted patients were predominantly elderly and retirees. It is important to note that consumers do not need to pay a fee to enter into a contract arrangement. The contract arrangement system operates as part of the essential public health package funded by the government. Those who enter into contract arrangements still maintain freedom in their choice of providers. They are not obligated to abide by any conditions imposed by the contracts. Because “contract coverage” is one of the performance indicators of the essential public health package, CHCs often implement very aggressive strategies to incentivize residents to sign contracts. From the perspective of consumers, there is little to lose from entering into a contract with a CHC (29).

Our study revealed that consumer satisfaction with the contract arrangements fell short of expectations. We found that only 26.7% of the study participants obtained a summed satisfaction score of higher than 24, exceeding the neutral total. Although over 85% of the respondents were satisfied with the financial benefits of discounts in medical services, only slightly more than half of our study participants were satisfied with their entitlements for prioritized medical appointments and referrals and less than one third were satisfied with hospitals at home and telemedicine services. Specialist and hospital services can become more efficient and more effective by liaising with primary care workers (28). However, it is not possible for GPs to fulfill such a role without the trust and acceptance of consumers.

However, there are some positive signs. We found in this study that 79% of respondents were satisfied with the free prescription services for chronic diseases under the contract arrangements. This may ease some of the burdens on overcrowded hospitals (33). Further evidence needs to be gathered to support such a claim. In theory, the better management of chronic conditions through primary care is critical, not only to improve the quality of life of patients but also to reduce preventable hospital admissions (28). GPs are best positioned to manage chronic conditions as they can establish a long-term continuing relationship with their patients and they understand the multiple needs of their patients best (34). Interestingly, less than half the respondents in our study could name their contracting doctors. Liu et al. (35) found that patients in China favor a concept of continuity centered around team efforts facilitated by information technologies.

The Chinese government is determined to continue to expand the initiative of contract arrangements with primary care. Our study shows that consumers' lack of awareness and understanding of contract entitlements may jeopardize such an effort. The findings have some policy implications. Firstly, the CHC approach to entering into contracts with community residents does not provide any mechanism or incentives to ensure the effective provision and materialization of contract entitlements. Secondly, contracting by itself is not the ultimate goal. A systems approach is needed to enable contract arrangements to serve as an instrument to improve the continuity and coordination of patient care. Thirdly, more radical system restructuring in China, such as the introduction of a gatekeeping mechanism through primary care, can be hindered by the low confidence from the government and trust from the public (27). Finally, the fragmentations in policy development across sectors in China need to be addressed (36) so that various incentives can be better aligned (37) and public trust in primary care can be regained (15). Unlike many developed countries, where a “gatekeeper” role has been assigned to GPs through social health insurance policies (38, 39), the Chinese system currently depends on voluntary contract arrangements. A systems approach is needed to achieve the policy goals of the contract arrangement system. These will include, but are not limited to, a strong commitment from the government, better aligned policies across sectors, the strong capacity of GPs, and public endorsement and support (40, 41).

There are several limitations in this study. We assessed patient satisfaction with the contract entitlements, but did not evaluate its impacts on the entire healthcare delivery system. Further studies are needed to systematically examine the impacts of the contracting system. The study adopted a cross-sectional design, which prevents causal conclusions. The study sample was drawn from Hangzhou, one of the most developed regions in China. Any attempts to extrapolate the findings need to be cautious.

## Conclusions

Contract arrangements for community residents with local primary care institutions represent the Chinese government's effort to reverse the hospital-dominant system in healthcare service delivery. Despite the rapid expansion of contracting rates, consumer awareness and understanding of contract entitlements is low, according to the findings of this study. Higher awareness, better perceived health, and living with chronic conditions are significant predictors of higher levels of satisfaction with the contract arrangements. Although the current system may bring certain benefits to consumers living with chronic conditions, a better design of contract arrangements, if not a fundamental change in the health care system, will be required to further advance the contracting system. Improving consumers' awareness and understanding of contract entitlements may help, but alone it is not enough.

## Data Availability Statement

The raw data supporting the conclusions of this article will be made available by the authors, without undue reservation.

## Ethics Statement

Ethics approval for the study protocol was obtained from the Hangzhou Normal University (Reference Number 20190070). Written informed consent for participation was not required for this study in accordance with the national legislation and the institutional requirements.

## Author Contributions

JR and CL contributed to the conceptualization and methodological design. LR, MH, and XQ contributed to the collection and analysis of data. LR, JR, and CL contributed to the interpretation of the results and the drafting of the manuscript. All authors mentioned contributed to technical enrichment, the writing and also reviewed, and then approved the final manuscript.

## Funding

This work was supported by National Natural Science Foundation of China (71874047) and Basic Public Welfare Research Program of Zhejiang Province (LGF21G030003).

## Conflict of Interest

The authors declare that the research was conducted in the absence of any commercial or financial relationships that could be construed as a potential conflict of interest.

## Publisher's Note

All claims expressed in this article are solely those of the authors and do not necessarily represent those of their affiliated organizations, or those of the publisher, the editors and the reviewers. Any product that may be evaluated in this article, or claim that may be made by its manufacturer, is not guaranteed or endorsed by the publisher.
